# Sinusoidal Extremely Low-Frequency Electromagnetic Stimulation (ELF-EMS) Promotes Angiogenesis In Vitro

**DOI:** 10.3390/biomedicines13061490

**Published:** 2025-06-17

**Authors:** Lena Perez Font, Amanda Moya-Gomez, Hannelore Kemps, Ivo Lambrichts, Jean-Michel Rigo, Bert Brône, Annelies Bronckaers

**Affiliations:** 1 UHasselt-Hasselt University, Faculty of Medicine, and Life Sciences, BIOMED, Agoralaan C, 3590 Diepenbeek, Belgium; perez.lena84@gmail.com (L.P.F.); amanda.moyagomez@uhasselt.be (A.M.-G.); ivo.lambrichts@uhasselt.be (I.L.); jeanmichel.rigo@uhasselt.be (J.-M.R.); bert.brone@uhasselt.be (B.B.); 2 Centro Nacional de Electromagnetismo Aplicado, Universidad de Oriente, Santiago de Cuba 90400, Cuba; 3 Center for Molecular and Vascular Biology, Department of Cardiovascular Sciences, KU Leuven, Herestraat 49, 3000 Leuven, Belgium; hannelore.kemps@kuleuven.be

**Keywords:** extremely low-frequency electromagnetic stimulation, angiogenesis, nitric oxide, endothelial cell

## Abstract

**Background/Objectives**: Angiogenesis is the multistep process of the formation of new blood vessels. It is beneficial in scenarios that require tissue repair and regeneration, such as wound healing, bone fracture repair, and recovery from ischemic injuries like stroke, where new blood vessel formation restores oxygen and nutrient supply to damaged areas. Extremely low-frequency electromagnetic stimulation (ELF-EMS), which involves electromagnetic fields in the frequency range of 0–300 Hz, have been shown to reduce ischemic stroke volume by improving cerebral blood flow and recovery effects that are dependent on eNOS. Based on previous results, we herein explore the effects of ELF-EMS treatment (13.5 mT/10 and 60 Hz) on the activation of angiogenic processes in vitro in homeostatic conditions. **Methods**: Using human microvascular endothelial cells (HMEC-1), we studied cell proliferation, migration, and tube formation in vitro, as well as nitric oxide production and the effect of calcium and nitric oxide (NO) on these processes. Moreover, blood vessel formation was studied using a chicken chorioallantoic membrane (CAM) assay. **Results**: Our results showed that ELF-EMS increases proliferation, tube formation, and both the migration and transmigration of these cells, the latter of which was mediated via NO. In turn, calcium inhibition decreased ELF-EMF-induced NO production. Furthermore, ELF-EMS significantly increased blood vessel formation in the CAM assay. **Conclusions**: Our results indicated that ELF-EMS exposure (13.5 mT/10 and 60 Hz) significantly induces angiogenesis in vitro and in ovo, underscoring its potential application in the treatment of conditions characterized by insufficient blood supply.

## 1. Introduction

Angiogenesis—the growth of new vascular structures from existing blood vessels—is a multistep process that involves the activation, proliferation, migration, and tube formation of endothelial cells (ECs) [1]. It is a complex process wherein various growth factors, cytokines, enzymes, compounds of the extracellular matrix, and intracellular signaling molecules such as nitric oxide (NO) play an important role. NO, produced by endothelial nitric oxide synthase (eNOS), activates soluble guanylate cyclase (sGC) to generate cyclic guanosine monophosphate (cGMP), a second messenger that regulates downstream signaling. This pathway is tightly linked to the vascular endothelial growth factor (VEGF), as the VEGF stimulates eNOS to produce NO, creating a positive feedback loop that amplifies angiogenesis [2].

Impaired angiogenesis contributes to the pathogenesis of several diseases, such as neurodegenerative disorders, hypertensive vascular dysfunction, and cardiovascular and cerebrovascular ischemia. During ischemic stroke, angiogenesis plays an important role in promoting nerve regeneration and functional recovery; therefore, promoting angiogenesis is a useful strategy to enhance stroke recovery [3,4].

Nowadays, therapeutic approaches include growth factor-based therapies including the use of the vascular endothelial growth factor (VEGF) [5]. Recent studies have shown that the VEGF not only increases angiogenesis but also enhances vascular permeability, which is associated with edema formation and increased hemorrhagic risk, which may have fatal consequences [6]. Other therapeutic approaches include stem cell transplantation [1,7,8], nanoparticle-based approaches with a proven ability to boost angiogenesis and decrease vascular permeability [9], gene therapy with the multi-isoform VEGF [10], other growth factors like the fibroblast growth factor (FGF) [11], erythropoietin (EPO) [12], microRNA therapy involving miR-126 and miR-221-3p, the effects of which promote angiogenesis via VEGF and AKT/eNOS signaling, respectively [13,14], and extremely low-frequency electromagnetic stimulation (ELF-EMS) [15,16]. The effectiveness of proangiogenic therapies is often limited by issues related to the appropriate dosing and retention of therapeutic agents at the target site [17]. ELF-EMS, however, has emerged as a promising alternative or adjunct to traditional proangiogenic therapies with the potential to enhance vascular perfusion and promote angiogenesis through several mechanisms [18,19].

Previous research has demonstrated that ELF-EMS may promote angiogenesis by enhancing tube formation and increasing the proliferation of endothelial cells (ECs). Yet, the underlying molecular mechanisms involved and the effect of different ELF-EMS doses on inducing these angiogenic processes are unknown [20,21,22]. ELF-EMS has been shown to improve tissue repair in chronic wounds via protease matrix rearrangement, neo-angiogenesis, epithelialization, and VEGF signaling pathway activation, stimulating regenerative processes [23,24].

Other authors have reported the adverse effects of ELF-EMS on blood vessel formation, for example, a reduction in the expression of key angiogenic factors like the VEGF and transforming growth factor β1 (TGF-β1) in metastatic cell lines [25,26], as well as a reduction in blood vessel density in a murine model of angiogenic growth [27]. In contrast, we previously demonstrated that ELF-EMS enhances vascular perfusion by stimulating NO production via Akt-/endothelial nitric oxide synthase (eNOS) signaling and reduces the ischemic stroke volume in different permanent stroke models by increasing cerebral blood flow. Moreover, the beneficial effects of ELF-EMS after permanent stroke were lost after NO inhibition, indicating the indispensable role of ELF-EMS in modulating ECs responses [18,19]. Based on these results, we wanted to explore the effects of ELF-EMS specifically on angiogenesis, using normoxic conditions as a starting point. Considering also the contradictory results found within the literature, there is a need to deepen the understanding of the cellular response and the molecular mechanisms involved in ELF-EMS-induced ECs responses.

The impact of sinusoidal ELF-EMS on crucial steps of the angiogenic cascade (ECs proliferation, migration, and tube formation) was assessed in homeostatic conditions, as well as the involvement of the NO/eNOS signaling pathway. Finally, we evaluated the ability of ELF-EMS to induce the formation of fully functional blood vessels in the chicken chorioallantoic membrane (CAM) assay.

## 2. Materials and Methods

### 2.1. Specifications of ELF-EMS Exposure System

**Figure 1:**
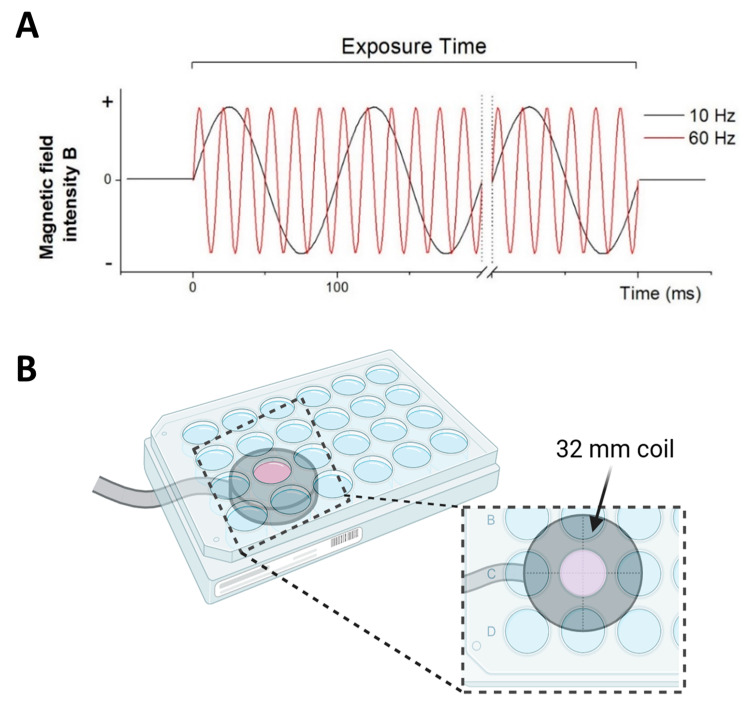
An illustration of the applied sinusoidal ELF-EMS. (**A**) Sinusoidal magnetic waves were generated continuously at either 10 or 60 Hz during the entire exposure time. (**B**) A schematic representation of the experimental setup for cell culture experiments: the plastic-enclosed coil is positioned beneath the culture plate, with the well(s) to be treated positioned over the center of the coil. The image was created using Servier Medical Art (https://smart.servier.com/), licensed under a Creative Commons Attribution 4.0 Unported License.

The application system to generate sinusoidal ELF-EMS was previously described in [12]. Briefly, ELF-EMS was generated using a coil (ferromagnetic core radius 16 mm; wire diameter 0.20 mm; 950 turns) connected to a Magnetic Stimulator NaK-02 manufactured by our collaborator from the Centro Nacional de Electromagnetismo Aplicado, Cuba. The resulting magnetic field showed a sinusoidal distribution as a function of time with an electromagnetic field strength of 13.5 mT and a non-pulsed sinusoidal frequency of 10 and 60 Hz (Figure 1A). Cell cultures were either stimulated or sham-stimulated (control) with the coil positioned below the targeted well (Figure 1B). For every plate size, the treated wells were positioned in the center of the coil. For the control group, the cell cultures were located above the coil without exposure to the electromagnetic field. For each experiment (n) with exposure of 60 Hz and 10 Hz, a control culture was sham-exposed (placed above the inductor with the device turned off).

### 2.2. In Vitro Angiogenic Assays

#### 2.2.1. Proliferation Assay

Human immortalized microvascular endothelial cells (HMEC-1) were used for all in vitro experiments and were cultured as previously described in [13]. To assess the effect of ELF-EMS on ECs proliferation, HMEC-1 cells were seeded in a 96-well plate (10,000 cells/well), and 24 h later, ELF-EMS or sham treatment was applied for 4 subsequent days, for 20 min per day. Cell viability was determined after the last treatment using the MTT (3-(4,5-dimethylthiazol-2-yl)-2,5-diphenyltetrazolium bromide, Sigma, St. Louis, MO, USA) assay as described in [28], which is commonly used as a cell proliferation assay [29,30]. Two wells were seeded each time per condition, and the results were averaged.

#### 2.2.2. Wound Healing Assay

Migration was measured first via the wound healing assay using a special culture Ibidi insert (Ibidi, Planegg/Martinsried, Germany). HMEC-1 (20,000 cells per well) were seeded, and 24 h later, the insert was removed, the medium was refreshed, and the cells were subjected to ELF-EMS with different levels of magnetic flux (2.5 mT, 4 mT, 13.5 mT, and 20 mT) and frequency (10 Hz and 60 Hz) for 20 min. After 24 h, the cells were fixed, stained with 0.1% cresyl violet (Sigma-Aldrich, Dorset, UK), and the area occupied in the wound by migrated cells was quantified. The values are presented in relative units that represent the fold change migration of the treated groups regarding the control group [31].

#### 2.2.3. Transwell Migration Assay

Transwell migration assay was performed using an 8 μm pore-size membrane separating the two chambers (Greiner Bio-One, Vilvoorde, Belgium). A total of 100,000 HMEC-1 cells were seeded in the upper compartment, while the lower compartment contained α-MEM complemented with 10% fetal bovine serum (FBS) as a chemoattractant. Three hours after seeding, the cells were subjected to ELF-EMS for 20 min. After 24 h, the cells that had transmigrated towards the lower chamber were fixed and stained with crystal violet for quantification. To analyze the implications of NO in ELF-EMS-induced ECs migration, HMEC-1 cells were preincubated for 15 min with the NOS inhibitor L-NMMA (1.5 mM, Tocris Bioscience, Bristol, UK) or the inducible NOS (iNOS) inhibitor 1400W (1.5 mM, Cayman Chemical, Ann Arbor, MI, USA). Lipopolysaccharide (LPS, 1 ng/mL, Sigma-Aldrich, St. Louis, MO, USA), a stimulator of iNOS production, was added as a positive control. Four images were taken for quantification with a Jenoptik ProgRes C3 (Jenoptik, Jena, AL, USA) camera coupled to an inverted microscope (Nikon Eclipse TS100, Tokyo, Japan) with a 100× magnification.

#### 2.2.4. Tube Formation Assay

In the tube formation assay, wells of an angiogenesis μ-slide (Ibidi, Martinsried, Germany) were coated with Matrigel (Corning, Bedford, MA, USA). After the solidification of the matrix, HMEC-1 were seeded (10,000 cells/well) and 1 h later, ELF-EMS (10 and 60 Hz) was applied for 20 min. After 6 h, pictures were taken and the number of nodes and total branching length were independently determined with the Angiogenesis Analyzer plugin (Image J v1.54m) [32,33] by a researcher blinded to the experimental conditions.

### 2.3. Quantification of Nitrite Production

Nitrite levels, as an indirect measure for NO production, were quantified using the Griess reaction system (Promega, Leiden, The Netherlands) according to the manufacturer’s instructions. HMEC-1 cells were preincubated or not with inhibitors L-NMMA (1.5 nM), 1400 W (1.5 mM), and intracellular Ca^2+^ chelator BAPTA-AM (Tocris Bioscience, Bristol, UK) (1 mM, 100 μM, 10 μM). Next, the cells were submitted to ELF-EMS (13.5 mT/10 Hz and 60 Hz, 20 min), and the medium was collected after 24 h for nitrite quantification.

### 2.4. Chorioallantoic Membrane Assay (CAM)

The angiogenic potential of ELF-EMS was measured with the CAM assay based on [28]. The CAM assay is a well-established angiogenesis model due to its abundant capillary network. It serves as a substitute for animal models and offers a natural setting for developing blood vessels [34]. Briefly, fertilized chicken eggs (*Gallus gallus*) were incubated for 3 days at 37 °C and constant humidity. On day 3 of embryonic development (E3), 3–4 mL of albumina was removed to detach the egg shell from the developing CAM, and a 1 cm^2^ window was opened in the shell, exposing the CAM. The window was covered with cellophane tape, and the eggs were returned to the incubator. At E4, the eggs were submitted to ELF-EMS (13.5 mT/10 and 60 Hz/20 min) for 4 days by placing the inductor on top of the window with the exposed CAM. Seven days after the first ELF-EMS exposure (E11), the CAM was removed, and the number of blood vessels (BVs) was assessed independently by two double-blinded researchers.

### 2.5. Statistical Analysis

Statistical analysis was performed with the GraphPad Prism 10 software (GraphPad, San Diego, CA, USA). The normal distribution was analyzed with a D’Agostino and Pearson normality test. For the wound healing assay, a repeated measurement ANOVA with a Bonferroni post-hoc test was used. When multiple groups were compared, a one-way ANOVA test with a Bonferroni Multiple Comparison post-hoc test was used for Gaussian distributed data or a Kruskall–Wallis test with Dune Multiple Comparison was otherwise used. The results are presented as means ± standard error of the mean (SEM). Differences were considered statistically significant when *p* < 0.05.

## 3. Results

### 3.1. ELF-EMS Stimulates Proliferation, Migration, and Tube Formation of HMEC-1 Cells

**Figure 2:**
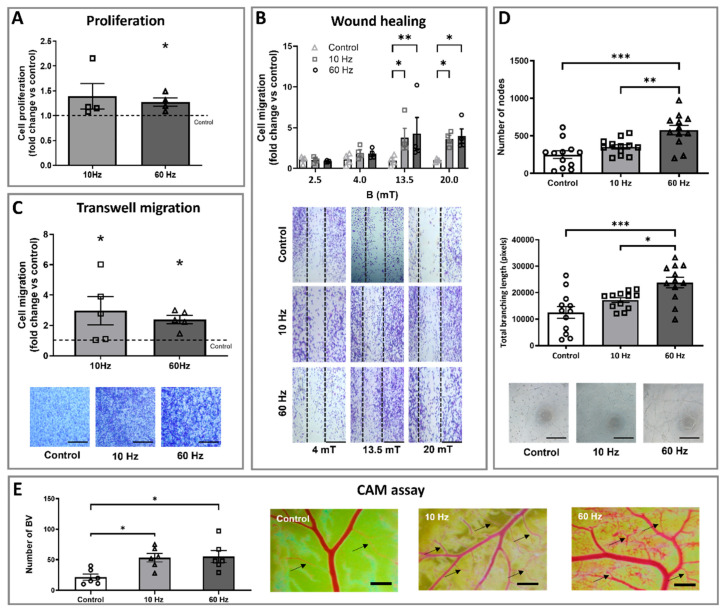
ELF-EMS exerts angiogenic properties both in vitro and in ovo. (**A**) The quantification of HMEC-1 proliferation. HMEC-1 were seeded and stimulated for 4 consecutive days with 13.5 mT at 10 or 60 Hz. Proliferation was assessed with the MTT assay (n = 4) on day 4 and normalized towards control conditions (dashed line). (**B**) Quantification and representative images showing HMEC-1 cell migration in the wound healing assay (4, 13.5, and 20 mT). The cells were seeded in an Ibidi insert, and the next day, after insert removal, the cells were stimulated with different doses of magnetic flux (2.5, 4, 13.5, and 20 mT) for 20 min. After 24 h, the number of cells that migrated into the cell-free space (as shown in the pictures) was measured (n = 4–5). Values were normalized towards control conditions (dashed line). Scale bars = 200 µm. (**C**) Quantification and representative images showing migrated cells in the transwell migration assay. HMEC-1 cells were seeded onto an 8 µm insert that was placed onto a well containing medium with 10% FBS. After 4 h, ELF-EMS was applied, and at 24 h, the number of migrated cells was assessed (n = 5). Values were normalized towards control conditions (dashed line). Scale bars = 200 µm. (**D**) The quantification of nodes and branching length and representative images showing HMEC-1 tube formation. Cells were seeded onto Matrigel. After 1 h, ELF-EMS was applied, and the number of formed nodes and the total branching length were assessed (n = 12) after 6 h. Scale bars = 200 µm (**E**) Quantification and representative images of the effect of ELF-EMS on the number of blood vessels in the CAM assay of the control and ELF-EMS conditions. The arrows indicate various blood capillaries. At E4, fertilized chicken eggs were subjected for 20 min to ELF-EMS (13.5 mT/10 or 60 Hz) for 4 days, and the number of capillary blood vessels was assessed seven days after the first ELF-EMS exposure (n = 6/group). Scale bars = 1 mm. Control groups were sham-exposed to the ELF-EMS equipment in all experiments for 20 min. Values are expressed as the mean ± SEM. (* *p* ≤ 0.05, ** *p* ≤ 0.01, *** *p* ≤ 0.001 as calculated by a Kruskall–Wallis test with Dune Multiple Comparisons for proliferation and transwell assay, one-way ANOVA with a Bonferroni Multiple Comparison Test for the tube formation and CAM assay, and repeated measurement ANOVA with Bonferroni a post-hoc test for the wound healing experiment.

Since ECs proliferation represents one of the key steps in angiogenesis, the effect of ELF-EMS on the rate of HMEC-1 cell growth was assessed. Four stimulations of 13.5 mT/60 Hz ELF-EMS treatment significantly increased the proliferation rate of HMEC-1 compared to the control group. There were no statistically significant differences between the 10 and 60 Hz, nor between the control group and 10 Hz (Figure 2A).

Besides proliferation, cell migration is a crucial step in blood vessel formation. Because differences in ELF-EMS-induced effects have been described depending on the magnetic induction [27,35], several values were included in this experiment to make a dose–response curve. The motility of HMEC-1 cells improved with the increasing intensity of the magnetic flux for both treated groups (10 and 60 Hz) compared to the control, with no evident effect for the lower inductions (2.5 and 4 mT). For both frequencies, a statistically significantly increased migration was observed for 13.5 and 20 mT. The maximal migration was reached at 13.5 mT, and the EC50 was estimated at ± 5.58 mT for both 10 and 60 Hz (Figure 2B). Based on these results, 13.5 mT was chosen as the magnetic flux value for the following experiments.

The effect of ELF-EMS on HMEC-1 migration was further assessed via the transwell migration assay. Cell migration showed a statistically significant increase by 3.0-fold and 2.4-fold when submitted to 10 Hz and 60 Hz stimulation, respectively (Figure 2C). Interestingly, treatment with 13.5 mT/60 Hz significantly induced HMEC-1 tube formation (Figure 2D). The total number of nodes increased 2.32 times, while the total branching length increased 2.9-fold compared to the control, with no effect observed for 10 Hz.

### 3.2. ELF-EMS Enhances In Ovo Angiogenesis

The CAM assay was performed to provide more insights into the angiogenic properties of ELF-EMS to induce the formation of new functional blood vessels in an in vivo model (Figure 2E). The number of BVs in both 10 and 60 Hz ELF-EMS-treated groups was statistically significantly higher (2.6- and 2.5-fold increase, respectively) than the control group, indicating that ELF-EMS can induce blood vessel development. No difference between the two treatment groups was found.

### 3.3. ELF-EMS-Induced HMEC-1 Migration Is Mediated by NOS

**Figure 3:**
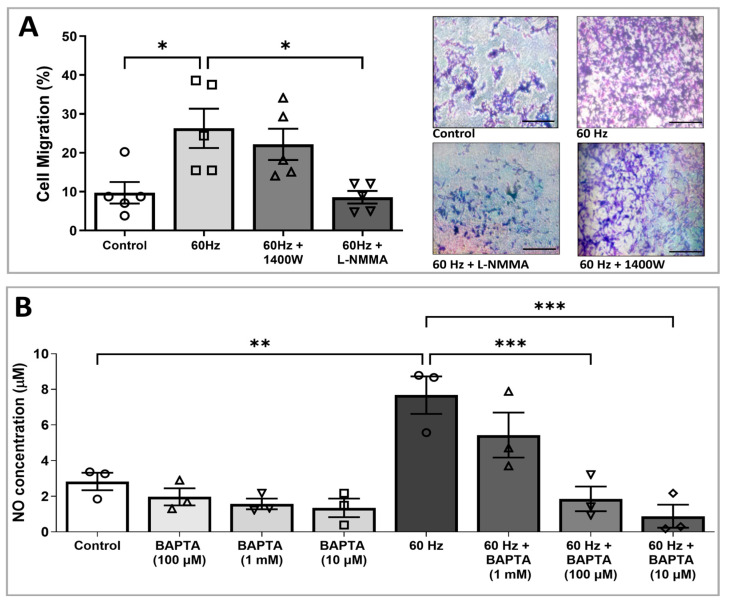
ELF-EMS-induced ECs migration is NOS-dependent, and calcium is involved in ELF-EMS-induced NO production. (**A**) Quantification and representative images of the effect of general NOS and iNOS inhibitors on the ELF-EMS-induced HMEC-1 cell migration. HMEC-1 cells were transferred onto transwell inserts and treated with NOS, iNOS inhibitors, and/or ELF-EMS (13.5 mT, 60 Hz). After 24 h, the number of migrated cells was assessed (n = 5). Scale bars = 200 µm. (**B**) Quantification of the effect of the Ca^2+^ chelator BAPTA-AM (BAPTA) on NO production of HMEC-1 in the presence or absence of ELF-EMS. HMEC-1 cells were treated with ELF-EMS in combination with various concentrations of BAPTA-AM. After 24 h, NO production was assessed with the Griess assay (n = 3). Legend: L-NMMA: NG-Monomethyl-L-arginine acetate (Inhibitor of all NOS isoforms); 1400 W: *N*-[3-(aminomethyl) benzyl] acetamidine (inhibitor of the iNOS isoform); BAPTA-AM: 1,2-Bis(2-aminophenoxy)ethane-*N*,*N*,*N*′,*N*′-tetraacetic acid tetrakis(acetoxymethyl ester). Values are expressed as the mean ± SEM. * *p* ≤ 0.05, ** *p* ≤ 0.01, and *** *p* ≤ 0.001 as calculated with one-way ANOVA with a Bonferroni post-hoc test.

We previously demonstrated that ELF-EMS increases endothelial NO levels by stimulating eNOS signaling [18,19]. To study whether ELF-EMS-induced migration in vitro is eNOS dependent, we analyzed the effect of different NOS inhibitors on ELF-EMS-induced ECs migration in the transwell migration assay. ELF-EMS at 13.5 mT/60 Hz stimulated the migration of HMEC-1 cells. The addition of the pan-NOS inhibitor (L-NMMA, 1.5 mM) to the ELF-EMS-treated endothelial cells showed a statistically significant decrease in migration in comparison with the group that only received ELF-EMS (27.69% ± 6.52 vs. 60.01% ± 15.41, respectively, ** *p* ≤ 0.01) (Figure 3A,B). This indicates that ELF-EMS-induced ECs migration is mediated via NOS signaling. In contrast, there was no statistically significant decrease observed in the migration of HMEC-1 cells treated with ELF-EMS and the iNOS inhibitor (1400 W) compared to ELF-EMS treatment alone. Considering that neuronal NOS is not expressed by ECs and that ELF-EMS-induced migration was inhibited by the general NOS inhibitor L-NMMA but not by the specific iNOS inhibitor 1400 W, our results strongly suggest that ELF-EMS-induced ECs migration is mediated by eNOS-dependent NO signaling.

### 3.4. Increased NO Production Induced by ELF-EMS Is Mediated via Calcium

Since Ca^2+^ acts as a second messenger that modulates the activity of many mediators, including eNOS, we analyzed the involvement of this cation in ELF-EMS-induced NO production. The nitrite levels of HMEC-1 cells, incubated with the intracellular Ca^2+^ chelator BAPTA-AM, were similar to thecontrol cells that did not receive any treatment. When this chelator was added to the samples stimulated with 60 Hz, a dose-dependent decrease in NO concentration was observed. In the presence of 10 μM BAPTA-AM, ELF-EMF-induced NO production slightly decreased. The addition of 100 μM and 1 mM of BAPTA-AM resulted in a statistically significant decrease in ELF-EMS-induced NO production compared to ELF-EMS-treated HMEC-1 alone (1.99 ± 0.67 µM and 1.03 ± 0.67 µM vs. 7.50 ± 0.95 µM, respectively). These results suggest that the increase in NO by ELF-EMS (13.5 mT/60 Hz) is Ca^2+^-dependent.

## 4. Discussion

In this study, we presented an analysis of the angiogenic effect of sinusoidal ELF-EMS (13.5 mT/10 or 60 Hz/20 min) in vitro and in ovo under homeostatic conditions. We previously demonstrated that this ELF-EMS treatment regime improved the neurological outcome and decreased the infarct size in a permanent global ischemic stroke model, of which the beneficial effects reverted with NO inhibition [12]. ELF-EMS at 13.5 mT/60 Hz also increased NO production in vitro via eNOS signaling in endothelial cells, and the activation of this pathway was responsible for ELF-EMS-induced vasodilation [19]. Based on these results, we wanted to explore whether these beneficial effects of ELF-EMS on stroke outcome can also be attributed to stimulation of angiogenesis. Therefore, we investigated the effect of ELF-EMS on crucial angiogenic processes, including proliferation, migration, and tube formation of HMEC-1 cells, but under homeostatic conditions as a preliminary approach.

Four applications for 20 min with 60 Hz favored the growth of ECs in vitro, indicating a direct activation of one of the first steps in the angiogenic cascade. Similar effects on proliferation have been reported by Katsir et al. [35], who observed an increase in the number of chicken embryo fibroblasts exposed to ELF-EMS (0.7 mT/100 Hz) for 24 h. McKay et al. described the activation of ECs proliferation and changes in human microvascular perfusion (increase and decrease) in the range of 0.5 mT to 20 mT with frequencies from 1 Hz to 72 Hz [36]. It is not specified whether the specified doses are measured on the inductor or in the working volume, and sometimes the wave type used and the time of the application are omitted, making it impossible to conduct an accurate comparison. In these studies, there were no dose–response curves provided, but all studies concurred in stating that ELF-EMS induced ECs proliferation [37].

Monache et al. [27] reported an increase in the proliferation and expression of vascular endothelial growth factor receptor 2 (VEGFR2) in human umbilical vein endothelial cells (HUVECs) after applying sinusoidal ELF-EMS (1 mT/50 Hz) for 6 and 12 h. Conversely, prolonging the duration of ELF-EMS treatment to 24 h resulted in the inhibition of HUVEC growth. This is in contrast with our results, where ELF-EMS was only applied for 20 min at 13.5 mT in 10 or 60 Hz. Moreover, pulsed EMF (PEMF) (15 Hz 1.8 mT) accelerated the proliferation and migration of rat cardiac microvascular ECs [38], while treatment with PEMF (2.25 mT/50 Hz/15 min) for 3 consecutive days increased HUVEC proliferation, but did not affect their migration [39].

However, our treatment significantly increased HMEC-1 migration as observed in the transwell migration and wound healing assays with a single application of ELF-EMS for both tested doses (13.5 mT/10 and 60 Hz). For the wound healing assay, other doses were applied to define the best response regarding migration and to assess the dose-dependent correlation. In comparison with other tested intensities, 13.5 mT induced the strongest endothelial response. It was even observed that by increasing the intensity up to 20 mT, there was a discrete decrease in migration compared to the 13.5 mT condition.

Migration entails fast membrane-to-cytoskeleton signaling, actin cytoskeleton remodeling, and adhesion dynamics, which involve integrin–extracellular matrix interactions and metalloproteinases (MPP secretion) [40]. ELF-EMF stimulation (50 Hz, 1 mT) promoted keratinocyte migration during wound healing by accelerating the early expression of IL-1β and MMP-9 production. Moreover, Akt and ERK pathways seem to mediate this effect [41], and we have previously demonstrated that our 60 Hz treatment scheme enhanced Akt activation [19]. On the other hand, proliferation is a slower process that depends on transcriptional regulation and the cell cycle [42]. Multiple sessions of 60 Hz-ELF-EMS may induce cumulative ERK activation and accelerate G1/S transition, as observed in endothelial cells under similar conditions but with longer exposures [43]. Considering that the energy of electromagnetic waves depends, among other things, on their frequency [44], it is possible that 60 Hz carries more energy compared to lower frequencies like 10 Hz, which may explain why the 60 Hz treatment may be sufficient to trigger cellular responses that promote proliferation. However, more insights into the effect of short-term ELF-EMS on cell cycle distribution are needed to fully comprehend its effect on proliferation.

Tube formation was also stimulated by ELF-EMS treatment: 60 Hz significantly induced the number of tubes, while 10 Hz was not statistically significant compared to the control. Previous studies reported that ELF-EMS of 1 mT intensity for 1, 6, and 12 h promoted HUVECs proliferation, motility, and tubule formation and that VEGFR2 (KDR/Flk-1) was involved in the angiogenic response of HUVECs to ELF-EMS [35], whereas the same author reported that 2 mT with the same experimental conditions induced a contrary effect. This apparent controversy is not surprising because it has been extensively demonstrated that the amplitude, frequency, and exposure pattern of ELF-EMS can significantly influence its biological effects [45].

To verify the angiogenic potential of ELF-EMS in an in vivo set-up, we quantified ELF-EMS-induced angiogenesis in the CAM model. Similar to the tubulogenic process, the 60 Hz treatment produced the largest angiogenic response. In this CAM assay, some studies reported the inhibitory effect of ELF-EMS [46,47,48], or even no effect at all [49]. Different settings are described, with none of them near the intensity and frequency proposed in this paper, and using longer exposure times. In some of these studies, the combined effect of ELF-EMS with an antiangiogenic agent enhances the overall antiangiogenic effect [48], indicating that the use of ELF-EMS in situations where homeostasis is compromised should be further explored. The different effects that ELF-EMS can possess are not only dependent on its parameters (frequency, duration, and magnetic flux density), but also the studied cell type [46]: for example, the same ELF-EMS settings have inhibitory effects on microglia migration [50]. Moreover, studies by Berg et al. have found different effects on angiogenesis using the same PEMF settings in normal cells compared to cancer cells, indicating that the effect of the PEMF is also related to the physiological state of cells [15,51].

In vivo, it has been reported that ELF-EMS augmented CD31^hii^ Endomucin^hi^ endothelial cells in a mouse model of postmenopausal osteoporosis [52], significantly increased wound healing in a hindlimb ischemia model [53], and enhanced the number and function of circulating endothelial precursors in rats with ischemia/reperfusion injury [54]. Interestingly, the protective effect of ELF-EMS in a focal permanent ischemic stroke model was related to the augmentation of cerebral collateral blood flow towards the infarcted area [19]. Since the effect on cerebral blood flow was only studied 1 and 24 h after stroke induction, the potential role of blood vessel formation in ELF-EMS-mediated effects on the cerebral vasculature should be explored at later time points, since angiogenesis can only be observed 14 to 28 days after stroke onset [55].

Moreover, our study demonstrates that NO, an important signaling molecule in activating ECs during angiogenesis, is a key player in the ELF-EMS-induced effects of ECs in vitro. More specifically, the NOS inhibitor L-NMMA inhibited ELF-EMS-induced ECs migration. These findings are in line with our previous results, where the addition of this inhibitor suppressed the NO production of HMEC-1 cells [19]. Other studies have demonstrated that the administration of pan-NOS inhibitor L-NAME prevents blood flow induced by the PEMF in healthy rat brains [56], and reduces ELF-EMS-induced blood flow enhancements in both the hind limb and brain of healthy mice [19]. In stroke models, NOS inhibition increases lesion size, preventing the beneficial effects of the ELF-EMS [18]. In previous studies, we have also shown that ELF-EMS activates eNOS by inducing the phosphorylation of Ser1177 [19]. This phosphorylation site is the target of many signaling molecules such as phosphoinositide 3-kinase, adenylate cyclase pathways, and shear stress. The phosphorylation of eNOS and Akt has been detected after EMF in a mouse hind limb ischemia [53,54]. Moreover, our treatment proved to increase cGMP, downstream signaling molecules in the NO cascade, which is directly related to migration, proliferation, and tube formation [19].

One of the most studied activation proteins of eNOS is Ca^2+^-dependent calmodulin (CaM). An increase in cytoplasmic calcium levels activates CaM, which binds to eNOS to promote the alignment of the oxygenase and reductase domains of eNOS, leading to more efficient NO synthesis. In addition, CaM can activate CaM kinase II, which phosphorylates eNOS on Ser1177. Based on this, we investigated whether Ca^2+^ is a mediator of ELF-EMS-induced NO production. Both nNOS and eNOS isozymes can be activated by CaM, whose activity depends on Ca^2+^. Our results showed that the addition of the Ca^2+^ chelator BAPTA-AM dose-dependently inhibited ELF-EMS-induced NO secretion in HMEC-1 cells [57].

The interaction of Ca^2+^ with EMFs has been previously addressed by other authors. In this sense, Blackman et al. [58] demonstrated that ELF-EMS (1–120 Hz) altered the influx and efflux (from intracellular stores) of Ca^2+^ ions in brain tissue ex vivo. Also, Santoro et al. [59] showed in lymphoid cells that exposure to ELF-EMS of 2 mT/50 Hz changes the plasma membrane and induces reorganization of the cytoskeleton. Moreover, ELF-EMS has been shown to enhance the activity of voltage-gated calcium channels [15,60]. It is generally accepted that the plasma membrane surface is the site of interaction of ELF-EMS and that some of the effects are dependent on Ca^2+^ regulation. In addition, Ca^2+^ is considered to be one of the main factors involved in the conversion of ELF-EMS signals into biological signals [61]. In this study, we observed that ELF-EMS-induced NO production is Ca^2+^ dependent, suggesting a direct effect of ELF-EMS on intracellular Ca^2+^, which could be linked to increased eNOS/NO signaling and, consequently, angiogenesis processes.

## 5. Conclusions

In this study, we proved that sinusoidal ELF-EMS at the doses tested (13.5 mT/10 and 60 Hz) induces several steps in the angiogenic process, namely proliferation, migration, and tube formation in vitro and blood vessel formation in ovo. Moreover, we found that the inhibition of NO decreases ELF-EMS-induced ECs migration and that the effect of ELF-EMS on NO production is linked to intracellular Ca^2+^. Our results shed some light on the mechanisms involved in the angiogenic effects of ELF-EMS. However, further research is essential to fully elucidate its mode of action in promoting angiogenesis, paving the way for its therapeutic application. It is important to highlight that this study did not investigate the effects of ELF-EMS on angiogenesis during ischemic conditions, which should be further explored in future investigations.

## Data Availability

The original contributions presented in this study are included in the article. Further inquiries can be directed to the corresponding author.

## Abbreviations

The following abbreviations are used in this manuscript:

ELF-EMS	extremely low-frequency electromagnetic stimulation
HMEC-1	human microvascular endothelial cells
NO	nitric oxide
CAM	chorioallantois membrane
VEGF	vascular endothelial growth factor
ECs	endothelial cells
eNOS	endothelial nitric oxide synthase
MTT	3-(4,5-dimethylthiazol-2-yl)-2,5-diphenyltetrazolium bromide
FBS	fetal bovine serum
iNOS	inducible nitric oxide synthase
nNOS	neural nitric oxide synthase
L-NMMA	NG-Monomethyl-L-arginine acetate
LPS	lipopolysaccharide
BAPTA-AM	1,2-bis(2-aminophenoxy)ethane-*N*,*N*,*N*′,*N*′-tetraacetic acid tetrakis(acetoxymethyl ester)
SEM	standard error of the mean
BVs	blood vessels
PEMF	pulsed electromagnetic field
VEGFR2	vascular endothelial growth factor receptor 2
HUVECs	human umbilical vein endothelial cells
L-NAME	LN G-Nitro arginine methyl ester
CaM	calmodulin

## References

[1] Carmeliet P., Jain R.K. (2000). Angiogenesis in cancer and other diseases. Nature.

[2] Li D.-Y., Gao S.-J., Sun J., Zhang L.-Q., Wu J.-Y., Song F.-H., Liu D.-Q., Zhou Y.-Q., Mei W. (2023). Targeting the nitric oxide/cGMP signaling pathway to treat chronic pain. Neural Regen Res..

[3] Fang J., Wang Z., Miao C. (2023). Angiogenesis after ischemic stroke. Acta Pharmacol. Sin..

[4] Ergul A., Alhusban A., Fagan S.C. (2012). Angiogenesis: A harmonized target for recovery after stroke. Stroke.

[5] White A.L., Bix G.J. (2023). VEGFA Isoforms as Pro-Angiogenic Therapeutics for Cerebrovascular Diseases. Biomolecules.

[6] Rust R. (2020). Insights into the dual role of angiogenesis following stroke. J. Cereb. Blood Flow Metab..

[7] Nagai N., Kawao N., Okada K., Okumoto K., Teramura T., Ueshima S., Umemura K., Matsuo O. (2010). Systemic transplantation of embryonic stem cells accelerates brain lesion decrease and angiogenesis. NeuroReport.

[8] Fan Y., Shen F., Frenzel T., Zhu W., Ye J., Liu J., Chen Y., Su H., Young W.L., Yang G.-Y.Y. (2010). Endothelial progenitor cell transplantation improves long-term stroke outcome in mice. Ann. Neurol..

[9] Wang G., Li Z., Wang G., Sun Q., Lin P., Wang Q., Zhang H., Wang Y., Zhang T., Cui F. (2024). Advances in Engineered Nanoparticles for the Treatment of Ischemic Stroke by Enhancing Angiogenesis. Int. J. Nanomed..

[10] Nakamura K., Henry T.D., Traverse J.H., Latter D.A., Mokadam N.A., Answini G.A., Williams A.R., Sun B.C., Burke C.R., Bakaeen F.G. (2024). Angiogenic gene therapy for refractory angina: Results of the EXACT phase 2 trial. Circ. Cardiovasc. Interv..

[11] Liu Y., Liu Y., Deng J., Li W., Nie X. (2021). Fibroblast growth factor in diabetic foot ulcer: Progress and therapeutic prospects. Front. Endocrinol..

[12] Zhu L., Yuan Q., Jing C., Sun L., Jiang L. (2024). Angiogenic responses are enhanced by recombinant human erythropoietin in a model of periventricular white matter damage of neonatal rats through EPOR-ERK1 signaling. J. Neuropathol. Exp. Neurol..

[13] Lyttle B.D., Vaughn A.E., Bardill J.R., Apte A., Gallagher L.T., Zgheib C., Liechty K.W. (2023). Effects of microRNAs on angiogenesis in diabetic wounds. Front. Med..

[14] Yin K.J., Hamblin M., Eugene Chen Y. (2015). Angiogenesis-regulating microRNAs and ischemic stroke. Curr. Vasc. Pharmacol..

[15] Peng L., Fu C., Wang L., Zhang Q., Liang Z., He C., Wei Q. (2021). The Effect of Pulsed Electromagnetic Fields on Angiogenesis. Bioelectromagnetics.

[16] Oladnabi M., Bagheri A., Kanavi M.R., Azadmehr A., Kianmehr A. (2019). Extremely low frequency-pulsed electromagnetic fields affect proangiogenic-related gene expression in retinal pigment epithelial cells. Iran. J. Basic Med. Sci..

[17] Eelen G., Treps L., Li X., Carmeliet P. (2020). Basic and Therapeutic Aspects of Angiogenesis Updated. Circ. Res..

[18] Font L.P., Cardonne M.M., Kemps H., Meesen R., Salmon O.F., González F.G., Lambrichts I., Rigo J.-M., Brône B., Bronckaers A. (2019). Non-pulsed Sinusoidal Electromagnetic Field Rescues Animals From Severe Ischemic Stroke via NO Activation. Front. Neurosci..

[19] Kemps H., Dessy C., Dumas L., Sonveaux P., Alders L., Van Broeckhoven J., Font L.P., Lambrichts S., Foulquier S., Hendrix S. (2022). Extremely low frequency electromagnetic stimulation reduces ischemic stroke volume by improving cerebral collateral blood flow. J. Cereb. Blood Flow Metab..

[20] Delle Monache S., Alessandro R., Iorio R., Gualtieri G., Colonna R. (2008). Extremely low frequency electromagnetic fields (ELF-EMFs) induce in vitro angiogenesis process in human endothelial cells. Bioelectromagnetics.

[21] Saliev T., Mustapova Z., Kulsharova G., Bulanin D., Mikhalovsky S. (2014). Therapeutic potential of electromagnetic fields for tissue engineering and wound healing. Cell Prolif..

[22] Patruno A., Tabrez S., Pesce M., Shakil S., Kamal M.A., Reale M. (2015). Effects of extremely low frequency electromagnetic field (ELF-EMF) on catalase, cytochrome P450 and nitric oxide synthase in erythro-leukemic cells. Life Sci..

[23] Pesce M., Patruno A., Speranza L., Reale M. (2013). Extremely low frequency electromagnetic field and wound healing: Implication of cytokines as biological mediators. Eur. Cytokine Netw..

[24] Gualdi G., Costantini E., Reale M., Amerio P. (2021). Wound Repair and Extremely Low Frequency-Electromagnetic Field: Insight from In Vitro Study and Potential Clinical Application. Int. J. Mol. Sci..

[25] Cios A., Ciepielak M., Lieto K., Matak D., Lewicki S., Palusińska M., Stankiewicz W., Szymański Ł. (2024). Extremely low-frequency electromagnetic field (ELF-EMF) induced alterations in gene expression and cytokine secretion in clear cell renal carcinoma cells. Med. Pr. Work. Health Saf..

[26] Wang M.H., Chen K.W., Ni D.X., Fang H.J., Jang L.S., Chen C.H. (2021). Effect of extremely low frequency electromagnetic field parameters on the proliferation of human breast cancer. Electromagn. Biol. Med..

[27] Delle Monache S., Angelucci A., Sanità P., Iorio R., Bennato F., Mancini F., Gualtieri G., Colonna R.C. (2013). Inhibition of angiogenesis mediated by extremely low-frequency magnetic fields (ELF-MFs). PLoS ONE.

[28] Bronckaers A., Hilkens P., Fanton Y., Struys T., Gervois P., Politis C., Martens W., Lambrichts I. (2013). Angiogenic Properties of Human Dental Pulp Stem Cells. PLoS ONE.

[29] Kokilakanit P., Koontongkaew S., Utispan K. (2024). Nitric oxide has diverse effects on head and neck cancer cell proliferation and glycolysis. Biomed. Rep..

[30] Olomu I.N., Hoang V., Madhukar B.V. (2024). Low levels of nicotine and cotinine but not benzo[a]pyrene induce human trophoblast cell proliferation. Reprod. Toxicol..

[31] Rodriguez L.G., Wu X., Guan J.-L. (2005). Wound-Healing Assay. Cell Migration.

[32] Huuskes B.M., DeBuque R.J., Kerr P.G., Samuel C.S., Ricardo S.D. (2020). The Use of Live Cell Imaging and Automated Image Analysis to Assist With Determining Optimal Parameters for Angiogenic Assay in vitro. Front. Cell Dev. Biol..

[33] Proust R., Ponsen A.-C., Rouffiac V., Schenowitz C., Montespan F., Roux K.S.-L., De Leeuw F., Laplace-Builhé C., Mauduit P., Carosella E.D. (2020). Cord blood-endothelial colony forming cells are immunotolerated and participate at post-ischemic angiogenesis in an original dorsal chamber immunocompetent mouse model. Stem Cell Res. Ther..

[34] Shekatkar M., Kheur S., Deshpande S., Sakhare S., Sanap A., Kheur M., Bhonde R. (2024). Critical appraisal of the chorioallantoic membrane model for studying angiogenesis in preclinical research. Mol. Biol. Rep..

[35] Katsir G., Parola A.H. (1998). Enhanced proliferation caused by a low frequency weak magnetic field in chick embryo fibroblasts is suppressed by radical scavengers. Biochem. Biophys. Res. Commun..

[36] McKay J.C., Prato F.S., Thomas A.W. (2007). A literature review: The effects of magnetic field exposure on blood flow and blood vessels in the microvasculature. Bioelectromagnetics.

[37] Robertson J.A., Thomas A.W., Bureau Y., Prato F.S. (2007). The influence of extremely low frequency magnetic fields on cytoprotection and repair. Bioelectromagnetics.

[38] Li F., Yuan Y., Guo Y., Liu N., Jing D., Wang H., Guo W. (2015). Pulsed magnetic field accelerate proliferation and migration of cardiac microvascular endothelial cells. Bioelectromagnetics.

[39] Cheng Y., Qu Z., Fu X., Jiang Q., Fei J. (2017). Hydroxytyrosol contributes to cell proliferation and inhibits apoptosis in pulsed electromagnetic fields treated human umbilical vein endothelial cells in vitro. Mol. Med. Rep..

[40] Alonso-Matilla R., Provenzano P.P., Odde D.J. (2025). Physical principles and mechanisms of cell migration. Biol. Phys. Mech..

[41] Patruno A., Ferrone A., Costantini E., Franceschelli S., Pesce M., Speranza L., Amerio P., D’Angelo C., Felaco M., Grilli A. (2018). Extremely low-frequency electromagnetic fields accelerates wound healing modulating MMP-9 and inflammatory cytokines. Cell Prolif..

[42] Migliaccio G., Ferraro R., Wang Z., Cristini V., Dogra P., Caserta S. (2023). Exploring cell migration mechanisms in cancer: From wound healing assays to cellular automata models. Cancers.

[43] Zhang M., Li X., Bai L., Uchida K., Bai W., Wu B., Xu W., Zhu H., Huang H. (2013). Effects of low frequency electromagnetic field on proliferation of human epidermal stem cells: An in vitro study. Bioelectromagnetics.

[44] Hu C., Zuo H., Li Y. (2021). Effects of Radiofrequency Electromagnetic Radiation on Neurotransmitters in the Brain. Front. Public Health.

[45] Mukhopadhyay R., Paul S., Bhattacharya P., Patnaik R. (2021). Electromagnetic Field as a Treatment for Cerebral Ischemic Stroke. Front. Mol. Biosci..

[46] Ruggiero M., Bottaro D.P., Liguri G., Gulisano M., Peruzzi B., Pacini S. (2004). 0.2 T magnetic field inhibits angiogenesis in chick embryo chorioallantoic membrane. Bioelectromagnetics.

[47] Eydgahi S.M., Baharara J., Balanezhad S.Z., Samani M.A. (2015). The synergic effect of glycyrrhizic acid and low frequency electromagnetic field on angiogenesis in chick chorioallantoic membrane. Avicenna J. Phytomed..

[48] Sadoughi S.D., Zafar-Balanjad S., Baharara J., Nejhad Shahrokhabadi K.H., Sepehri Moghadam H., Rahbarian R. (2015). The effect of shoots of *Allium sativum* L., *Ferula assa-foetida* aqueous extract and low frequency electromagnetic field on angiogenesis in chick chorioallantoic membrane (in vivo). J. Med. Plants.

[49] Balanezhad S.Z., Parivar K., Baharara J., Kouchesfeh H.M., Ashraf A. (2010). The Effect of Extremely Low Frequency Electromagnetic Field on Angiogenesis. Res. J. Environ. Sci..

[50] Moya-Gómez A., Font L.P., Burlacu A., Alpizar Y.A., Cardonne M.M., Brône B., Bronckaers A. (2023). Extremely Low-Frequency Electromagnetic Stimulation (ELF-EMS) Improves Neurological Outcome and Reduces Microglial Reactivity in a Rodent Model of Global Transient Stroke. Int. J. Mol. Sci..

[51] Berg H., Günther B., Hilger I., Radeva M., Traitcheva N., Wollweber L. (2010). Bioelectromagnetic Field Effects on Cancer Cells and Mice Tumors. Electromagn. Biol. Med..

[52] Wang Q., Zhou J., Wang X., Xu Y., Liang Z., Gu X., He C. (2022). Coupling induction of osteogenesis and type H vessels by pulsed electromagnetic fields in ovariectomy-induced osteoporosis in mice. Bone.

[53] Li R.L., Huang J.J., Shi Y.Q., Hu A., Lu Z.Y., Weng L., Wang S.Q., Han Y.P., Zhang L., Hao C.N. (2015). Pulsed electromagnetic field improves postnatal neovascularization in response to hindlimb ischemia. Am. J. Transl. Res..

[54] Ma F., Li W., Li X., Tran B.H., Suguro R., Guan R., Hou C., Wang H., Zhang A., Zhu Y. (2016). Novel protective effects of pulsed electromagnetic field ischemia/reperfusion injury rats. Biosci. Rep..

[55] Kanazawa M., Takahashi T., Ishikawa M., Onodera O., Shimohata T., del Zoppo G.J. (2019). Angiogenesis in the ischemic core: A potential treatment target?. J. Cereb. Blood Flow Metab..

[56] Bragin D.E., Statom G.L., Hagberg S., Nemoto E.M. (2015). Increases in microvascular perfusion and tissue oxygenation via pulsed electromagnetic fields in the healthy rat brain. J. Neurosurg..

[57] Erkens R., Suvorava T., Kramer C.M., Diederich L.D., Kelm M., Cortese-Krott M.M. (2017). Modulation of Local and Systemic Heterocellular Communication by Mechanical Forces: A Role of Endothelial Nitric Oxide Synthase. Antioxid. Redox Signal..

[58] Blackman C.F., Benane S.G., House D.E., Joines W.T. (1985). Effects of ELF (1–120 Hz) and modulated (50 Hz) RF fields on the efflux of calcium ions from brain tissue in vitro. Bioelectromagnetics.

[59] Santoro N., Lisi A., Pozzi D., Pasquali E., Serafino A., Grimaldi S. (1997). Effect of extremely low frequency (ELF) magnetic field exposure on morphological and biophysical properties of human lymphoid cell line (Raji). Biochim. Biophys. Acta Mol. Cell Res..

[60] Pall M.L. (2013). Electromagnetic fields act via activation of voltage-gated calcium channels to produce beneficial or adverse effects. J. Cell. Mol. Med..

[61] Yuan J., Xin F., Jiang W. (2018). Underlying Signaling Pathways and Therapeutic Applications of Pulsed Electromagnetic Fields in Bone Repair. Cell. Physiol. Biochem..

